# Evaluating Perceived Probability of Threat-Relevant Outcomes and Temporal Orientation in Flying Phobia

**DOI:** 10.1371/journal.pone.0161272

**Published:** 2016-08-24

**Authors:** Elena Mavromoustakos, Gavin I. Clark, Adam J. Rock

**Affiliations:** School of Behavioural, Cognitive and Social Sciences, University of New England, Armidale, Australia; Nanjing Normal University, CHINA

## Abstract

Probability bias regarding threat-relevant outcomes has been demonstrated across anxiety disorders but has not been investigated in flying phobia. Individual temporal orientation (time perspective) may be hypothesised to influence estimates of negative outcomes occurring. The present study investigated whether probability bias could be demonstrated in flying phobia and whether probability estimates of negative flying events was predicted by time perspective. Sixty flying phobic and fifty-five non-flying-phobic adults were recruited to complete an online questionnaire. Participants completed the Flight Anxiety Scale, Probability Scale (measuring perceived probability of flying-negative events, general-negative and general positive events) and the Past-Negative, Future and Present-Hedonistic subscales of the Zimbardo Time Perspective Inventory (variables argued to predict mental travel forward and backward in time). The flying phobic group estimated the probability of flying negative and general negative events occurring as significantly higher than non-flying phobics. Past-Negative scores (positively) and Present-Hedonistic scores (negatively) predicted probability estimates of flying negative events. The Future Orientation subscale did not significantly predict probability estimates. This study is the first to demonstrate probability bias for threat-relevant outcomes in flying phobia. Results suggest that time perspective may influence perceived probability of threat-relevant outcomes but the nature of this relationship remains to be determined.

## Introduction

Flying phobia, also known as aviophobia and the fear of flying, is a highly prevalent situational specific phobia [1] with research suggesting that between 2.5% and 40.0% of the population experience anxiety related to flying on aeroplanes [2]. Individuals with flying phobia experience a number of fears regarding the flying experience [3] and experience significant anxiety when flying [4]. Consequently, flying phobics will often avoid or restrict their flying to the bare minimum, which may adversely impact on family, social and professional life [5].

The fear of flying has been found to be associated with the appraisal of both internal and external sources of threat [6]. External danger appraisals typically relate to negative flying outcomes (e.g. a plane malfunctioning, encountering turbulence or the plane crashing) and internal fears typically pertaining to the perceived consequences of anxiety sensations (e.g. loss of control [7]). The appraisal of the flying experience itself as dangerous may be conceptualised as irrational due to the relatively low probability of being involved in a flying-related accident compared to other forms of transport and other common daily activities [8]. Consequently, fears regarding imminent threat of external negative flying-related outcomes can be considered to reflect an overestimation of threat. It is noteworthy that the processes involved in the maintenance of flying phobia are poorly understood [6]. Thus, determining the mechanisms which lead to, and maintain, the overestimation of threat may facilitate a better understanding of flying phobia and how best to treat this common presenting problem.

Cognitive-behavioural conceptualisations of anxiety disorders suggest that the overestimation of the likelihood of a feared outcome contributes directly to the experience of anxiety [9]. Individuals with DSM-IV anxiety disorders [10] have been found to display a tendency to overestimate the contingency between fear-relevant stimuli and aversive outcomes, when such stimuli are relevant to the particular concerns for each disorder [11–13]. For example, individuals with obsessive-compulsive disorder (OCD) have been demonstrated to overestimate the threat posed by their intrusive thoughts [14], individuals with Social Anxiety Disorder (SAD) overestimate the cost of negative outcomes or evaluations by others [15], and individuals with Panic Disorder overestimated the potential consequences of physical sensations [16] relative to individuals without anxiety disorders. However, such an overestimation of threat has yet to be demonstrated in flying phobia.

### Probability Bias and Reasoning Processes

The appraisal and overestimation of threat in anxiety disorders is believed to be maintained by a variety of processes including domain-specific reasoning biases [17]. A reasoning bias occurs when thinking about the world favours particular conclusions in a systematic manner, across time and different contexts [13]. The role of reasoning biases in flying phobia has received little investigation.

*Probability bias* refers to the tendency to overestimate the likelihood of threat-relevant outcomes occurring in the future, specific to the concerns of a given psychological disorder [17]. Consequently, probability bias is typically demonstrated through obtaining probability estimates of fear-relevant outcomes occurring from individuals with a given disorder and comparing these with individuals who do not report the fears (i.e. the perceived threat) associated with this disorder. Probability bias has been demonstrated across a range of anxiety disorders [18–21]. For instance, Öst and Csatlos [21] found that participants who met diagnostic criteria for Claustrophobia estimated the probability of Claustrophobic-related negative events significantly higher than normal controls. They found no group differences for estimates of the probability of non-fear-relevant negative or positive events, thus, demonstrating a domain-specific probability bias. Similar findings in relation to Agoraphobic-related negative events have been reported for participants who met diagnostic criteria for Agoraphobia [20], and for individuals with SAD for threatening negative social events [19, 22]. Butler and Mathews [18] found that participants diagnosed with Generalised Anxiety Disorder (GAD) made higher estimates of the probability of negative events occurring than non-anxious controls when those events were predicted for themselves but not when these related to other people. Given such findings, it would seem reasonable to hypothesise that flying phobics would demonstrate a probability bias whereby they overestimate the likelihood of negative flying-related outcomes occurring relative to non-flying phobics.

The only study which has evaluated the perceived probability of negative flying-related outcomes in flying phobics found that estimates of aversive flying-related outcomes (e.g. the number of deaths as a result of commercial airline accidents) were equivalent across a group of flying and non-flying phobics [7]. However, it is notable that, unlike studies of probability bias described above, the questions and scenarios employed were not constructed to reflect a personally-relevant threatening outcome (i.e. personal likelihood of encountering an aversive outcome). As evidenced by Butler and Mathews [18], anxious individuals may make higher probability ratings for negative threatening events only when they are predicted for themselves. If a probability bias was demonstrated in flying phobia this would provide a clear onus for clinicians and researchers to measure and address this bias within the treatment of flying phobia.

A reasoning process which may contribute directly to the operation of probability bias in anxiety disorders is the *availability heuristic*, which refers to the tendency for the estimation of the likelihood of an event to be influenced by the relative availability and/or accessibility of related memories [23]. The *simulation heuristic* is a subtype of the availability heuristic and stipulates that expectancies are influenced by the relative ease of imagining mental simulations of related events [13]. The operation of these reasoning biases would suggest that greater availability and/or accessibility of a fear-related memory, or greater ease of imagining a future feared outcome, will lead to such fears being perceived to be more likely to occur [13]. The operation of the availability and simulation heuristics may be demonstrated in the findings of Carroll [24] who reported that instructing participants from the general population to imagine an event significantly increased expectations of the imagined event. Similarly, Sherman et al. [25] found that asking participants to imagine contracting a disease with ‘easy-to-imagine’ symptoms resulted in participants making significantly higher likelihood ratings of contracting the disease compared to a ‘difficult-to-imagine’ condition. In addition to these findings, there is evidence that episodic memory retrieval has been shown to influence the manner in which people imagine and simulate future events [26–27]. Consequently, the generation of future orientated mental imagery (*episodic future thought*; [28]) or retrieval of memories may directly impact on the perceived probability of future events occurring. Given the potential importance of future and past-orientated cognition in estimating the likelihood of fear-relevant future events occurring, an individual’s time orientation may be pertinent to understanding the operation of probability bias in flying phobia.

### Time Perspective and Probability Estimates

*Time perspective* describes an individual-differences variable, which accounts for the manner in which cognitive processes partition human experience into temporal frames [29], and has been defined as “the totality of the individual’s views of their psychological future and psychological past existing at a given time”[30] (p. 75). Time perspective can be assessed using the Zimbardo Time Perspective Inventory (ZTPI [29]), which measures individual differences in time orientations through the degree to which an individual is oriented towards five different temporal frames: *Past-Negative*, *Past-Positive*, *Present-Hedonistic*, *Present-Fatalistic* and *Future*. Each of these time orientations have been demonstrated to impact significantly on individual well-being, positive and negative affect and also upon predictions regarding one’s future [31]. Arnold et al. [32] adopted the ZTPI to investigate individual differences in temporal orientations and autonoetic consciousness as measured by an adapted version of the Memory Characteristics Questionnaire (MCQ [33]). *Autonoetic consciousness* refers to the ability to mentally represent subjective experiences in the past, present and future and, therefore, engage in ‘mental time travel’ [28]. Arnold et al. found that scores on the Present-Hedonistic subscale (which reflects an orientation towards present-focussed enjoyment, pleasure and excitement) and the Future subscale (which reflects an orientation towards planning for, and achievement of, future goals) positively predict feelings of re-experiencing the past and pre-experiencing potential future episodes. Therefore, these Future and Present-Hedonistic temporal orientations may predict the tendency to mentally time travel either backward or forward (i.e. autonoetic consciousness). Given that the retrieval of episodic memories and envisaging future events have both been implicated in increasing the perceived probability of such events occurring, Future and Present-Hedonistic orientations may hypothesised to be pertinent variables in individual estimates of the probability of threatening future events occurring.

Furthermore, the Past-Negative subscale of the ZTPI measures a negative orientation towards the past and the recall of aversive events. The Past-Negative subscale of the ZTPI contains a number of items, which speak to the function of memory and associated imagery, e.g., “*Painful past experiences keep being replayed in my mind*.” Given that memory retrieval has been shown to influence the manner in which people imagine and simulate future events [27], and the availability heuristic suggests that such memories impact directly upon predictions regarding the probability of future events, the Past-Negative subscale may also be pertinent in assessing factors that may influence the perceived likelihood of threatening future events occurring. It may, therefore, be hypothesised that individual orientation toward Future, Present-Hedonistic and Past-Negative temporal frames, as measured by the ZTPI, will be positively associated with perceptions of the probability of feared outcomes occurring.

### Links between Probability Bias, Flying Phobia and Time Perspective

Studies investigating probability bias in anxiety disorders typically instruct participants to imagine an event and, subsequently, rate the probability of the event occurring in the future [21]. Such research, therefore, ultimately encourages participants to generate mental representations of the described events, which would incorporate mental imagery. As described above, prior imagery instructions have been found to increase expectations of the imagined adverse event occurring in the external world in the future. Consequently, when individuals consider the probability of threat-relevant future events they may be hypothesised to form mental representations of future events as a means of evaluating the likelihood of their occurrence. In the case of flying phobia, it has been hypothesised that the formation of threat-related imagery may contribute directly to the perception of threat [34–35]. When evaluating the probability of events, the retrieval of similar episodic memories may also influence the manner in which people imagine future events [26]. Clark and Rock [35] proposed that the retrieval and mental reliving of aversive flight experiences (e.g. traumatic experiences or anxious states during previous plane flights) may serve to perpetuate anxiety and perceived threat in flying phobics. Indeed, the selective retrieval of threat-relevant outcomes has been demonstrated to contribute to individuals’ predications of the likelihood of negative outcomes in anxiety disorders [36]. If a probability bias exists in flying phobia, flying phobics will generally overestimate the likelihood of negative flying-related events, leading to an increase in the appraisal of danger associated with flying and higher levels of anxiety. It may, therefore, be hypothesised that individuals with greater temporal orientation towards past aversive experience (Past-Negative orientation) or who more readily form mental representations of future events (Future and Present-Hedonistic orientations) will perceive the likelihood of the occurrence of negative flying-related outcomes as more probable. To date, no research has measured time perspective in relation to flying phobia or the perceived probability of fear-relevant future events.

### The Present Study

The present study aimed to investigate whether participants with flying phobia would display a probability bias relative to non-flying phobics by reporting higher estimates of the probability of negative flying outcomes. Furthermore, the present study aimed to determine whether scores on the Past-Negative, Future and Present-Hedonistic subscales of the ZTPI predicted the estimated probability of negative flying outcomes.

Based on the findings of Öst and Csatlsos [21] it was hypothesised that: (1) flying phobics would rate the probability of negative flying events higher than non-flying phobics; (2) there would be no difference between flying and non-flying phobics on estimates of the probability of general negative events; and (3) there would be no difference between the estimates of flying and non-flying phobics regarding the probability of general positive events.

As established cognitive behavioural theory suggests that the overestimation of threat contributes directly to anxiety experienced in response to threat-relevant stimuli [9] it was hypothesised that: (4) probability estimates for the occurrence of negative flying events would be positively associated with anxiety experienced when flying. Finally, due to the findings that scores on the ZTPI subscales may predict autonoetic consciousness, and based on the arguments put forward by Clark and Rock [35], it was hypothesised that: (5) Past-Negative, Future and Present-Hedonistic scores would positively predict probability estimates for the occurrence of negative flying events.

## Method

### Participants

Two hundred and fifty-one participants commenced the online study. Potential participants were invited to complete the study if they reported that they experienced little or no anxiety when flying (as indicated by rating their flight anxiety as 0, 1 or 2 out of 10 on a visual analogue scale) or high levels of anxiety when flying (indicated by rating 8, 9 or 10 on a visual analogue scale). Selecting individuals for inclusion based on scoring at the upper or lower end of a single flight anxiety rating scale has been employed in a number of flying phobia studies [7]. Consequently, participants were excluded from the study if they scored 3–7 on a visual analogue scale measuring flying anxiety. Participants were also excluded if they were under the age of 18. One hundred and fifteen individuals who met the entry requirements participated in the study. The individuals in the high flying anxiety group and the low flying anxiety group will be labelled the *flying phobic* and *non-flying phobic* groups. Sixty flying phobics (mean age = 37.53 years, *SD =* 12.86) and 55 non-flying phobics (mean age = 32.64 years, *SD =* 13.47) participated.

Prior to commencing the study, a power analysis indicated that a minimum total sample of 77 participants was required, assuming an effect size (*f*^2^) of .15, a target power of .80, a critical value of .05 and three predictors. Participants were recruited through public information noticeboards, online public forums (e.g. Facebook pages) and social networking sites (e.g. fear of flying blogs and discussion boards) and students from the University of New England (who received course credit for participation).

### Measures

#### Visual Analogue Flight Anxiety Scale (VAFAS) [4]

The VAFAS [4] is a visual analogue scale which asks participants to indicate the extent to which they typically feel anxious when flying on a scale from 0 (“no flight anxiety”) to 10 (“terrified”). The scale is represented on a horizontal line, anchored by word descriptors at each end. Visual analogue scales have previously been employed to identify low and high anxiety fliers in a number of flying phobia studies [6] and are considered an appropriate screening instrument for identifying flying and non-flying phobics.

#### Flight Anxiety Situations Questionnaire (FAS)

The FAS [37] is a 32-item self-report inventory assessing anxiety experienced in different flight situations. The scale yields a total flight anxiety score as well as scores on three subscales: (1) an Anticipatory flight anxiety scale, comprised of 14 items related to anxiety experienced when anticipating a flight; (2) an In-Flight anxiety scale, comprised of 11 items related to anxiety experienced during a flight; and (3) a Generalised flight anxiety scale, comprised of seven items related to anxiety experienced with flying in general. Responses are made on a five-point scale, ranging from 1 (“no anxiety”) to 5 (“overwhelming anxiety”). The FAS has been found to have good internal consistency, with Cronbach’s alpha ranging from .85 to .96 [4, 37]. In the present study, Cronbach’s alpha for total FAS score and each subscale ranged from .88 to .99.

#### Probability Scale (PS)

The Probability Scale [21] is a 20-item scale designed to assess probability estimates for fear-relevant outcomes of individuals with Claustrophobia. The scale consists of three subscales describing: (1) Claustrophobic Negative events, comprised of eight items; (2) General Negative events, comprised of seven items (e.g. “*It is night-time and a thunderstorm*. *Lightning hits your house and your house catches fire”*); and (3) General Positive events, comprised of five items (e.g. *“You are out walking and find a 100-dollar note on the ground in front of you”*). Each subscale yields an average probability estimate for the type of future event described. Participants are instructed to “Imagine that you, unaccompanied, are in the situations described in the items. How probable do you think it is that the described event really will take place?” on a scale between 0% (“it does not happen at all”) and 100% (“completely certain that it happens”). For the purposes of this study, the eight Claustrophobic Negative events were replaced with eight flight-related negative events (e.g. *“You are in a plane taking off from a busy city airport*. *Five minutes after take-off your plane collides with a plane attempting to land”*) based on commonly reported fears of flying phobics [3, 7] and the flying scenarios described in the Articulated Thoughts during Simulated Situations utilised with flying phobics [38]. Thus, a *Flying Negative* subscale was created. Cronbach’s alpha for the present study was .92 for the Flying Negative items, .87 for General Negative items and .76 for General Positive items, suggesting good internal consistency in this sample.

#### The Zimbardo Time Perspective Inventory (ZTPI)

The ZTPI [29] is a quantitative measure of the psychological construct of time perspective. It asks participants to rate how characteristic a statement is of them on a 5-point Likert scale ranging from 1 (“uncharacteristic”) to 5 (“very characteristic”). It is composed of five factors pertinent to individual temporal profiles: (1) Past-Negative; (2) Past-Positive; (3) Present-Hedonistic; (4) Present-Fatalistic; and (5) Future. The present study utilised the Past-Negative, Present-Hedonistic and Future subscales due to the rationale described above. The ZTPI has been found to have good internal consistency, with Cronbach’s alpha ranging from .77 to .82 [29]. In the present study, Cronbach’s alpha was .85 for the Past-Negative subscale, .80 for the Present-Hedonistic subscale and .82 for the Future subscale.

### Procedure

Ethics approval was obtained from the Human Research Ethics Committee at the University of New England. Participants were recruited by placing study invitations to participate on public information noticeboards, online public forums, fear of flying online discussion boards and social networking sites. The questionnaires were compiled into an online survey utilising Qualtrics Research Suite software (Qualtrics, Provo, UT). Participants were directed to the study’s link on Qualtrics and asked to provide online implied consent (i.e. by clicking on the “Proceed to study” button underneath the consent to participate statements participants indicated their agreement to participate). Following this consent participants answered questions regarding demographic variables and background questions relating to their flying behaviours. Participants were, subsequently, screened for inclusion in the flying phobic and non-flying phobic groups by completing the VAFAS. Participants who scored at the upper end of the one-tailed visual analogue scale (8, 9 or 10) became the flying phobic group and those that scored at the lower end of the visual analogue scale (0, 1 or 2) became the non-flying phobic group. Both groups proceeded with the study and completed the FAS, PS and ZTPI. Participants who scored between 3 and 7 were thanked for their time and did not continue with the study.

## Results

### Participant Characteristics

Of the initial 251 participants, 54 (22%) scored between 3 and 7 on the VAFAS and 82 (33%) failed to complete the study and, thus, their data was excluded from the final data subjected to analysis (see S1 File). The use of the VAFAS to identify a flying phobic and non-flying phobic group was supported by the means on the FAS, which were similar to those obtained by Nousi et al. [4] in a sample of over 2000 flying phobics and over 1000 non-flying phobics. The flying phobic group in the current study recorded a mean total FAS score of 111.68 (*SD* = 19.76), which is higher than the reported norms for Nousi et al.’s flying phobic sample of 102.42 (*SD* = 22.48). The non-flying phobic group recorded a mean total FAS score of 40.80 (*SD* = 12.04), which is very close to the reported FAS norms for the general non-flying phobic population of 39.82 (*SD* = 11.92). This suggests that the grouping reflects a valid representation of individuals with high and low flying-related anxiety. Participant characteristics are presented in Table 1.

**Table 1:** Descriptive statistics for flying and non-flying phobics.

Group	Gender		Mean Age (*SD*)	No. of flights taken in past 12 months				
	Male	Female		0	1–2	3–5	6–10	10+
**Flying phobics**	8	52	37.53 (12.86)	15	27	10	5	3
**Non-flying phobics**	30	25	32.64 (13.47)	8	17	17	7	6

### Hypotheses 1–3: Group Differences on Probability Scale

**Fig 1:**
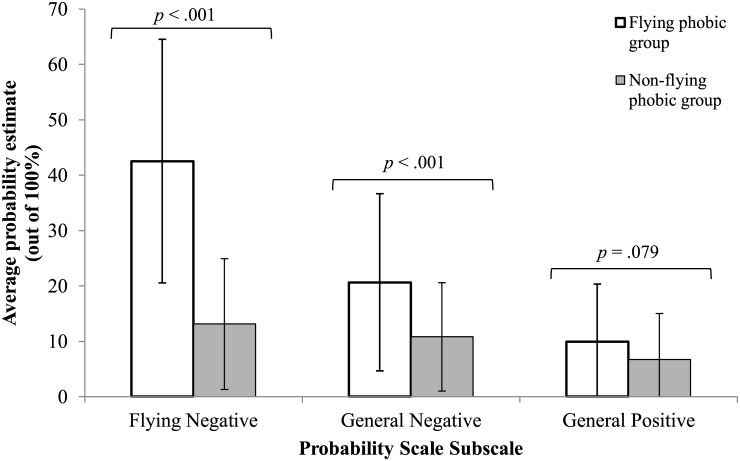
Group Differences on the Probability Scale subscales.

Independent samples *t*-tests [39] were conducted to explore group differences on the Flying Negative, General Negative and General Positive subscales of the PS. Means, standard deviations, *t*-test values and *p*-values are presented in Table 2. Results indicated that there were statistically significant group differences with the flying phobic group estimating the probability of the occurrence of Flying Negative and General Negative events as significantly higher than non-flying phobics. In order to account for the proportion of variance in the dependent variable accounted for by the independent variable (i.e. flying phobic versus non-flying-phobic) an eta squared (η^2^) was calculated [40]. A large difference was found between the means of the flying phobic and non-flying phobic group for Flying Negative outcomes (η^2^ = .406) and General Negative outcomes (η^2^ = .122). These results support Hypothesis 1, but did not support Hypothesis 2, which predicted that there would be no group differences for General Negative scores. There were no significant group differences for the estimated probability of General Positive events, thus, supporting Hypothesis 3. Group differences are represented visually in Fig 1.

**Table 2:** Mean, Standard Deviations (SD) and Independent t-values of FAS subscales for Flying and Non-flying Phobics.

	Non-flying phobics (*n* = 55)		Flying phobics *(n* = 60)			
	Mean	*SD*	Mean	*SD*	*t*(*df*)	*p*
**Flying Negative**	13.13	11.87	42.52	22.74	-8.79 (113)	.000*
**General Negative**	10.81	9.84	20.63	16.24	-3.96 (113)	.000*
**General Positive**	6.74	8.38	9.90	10.47	-1.77 (113)	.079

* *p* < .001

### Hypothesis 4: Probability Estimates for the Occurrence of Negative Flying Events would be Positively Associated with Anxiety Experienced when Flying

A series of Pearson’s correlations [39] were conducted to examine the association between Flying Negative scores and FAS scores (including total, anticipatory anxiety, in-flight anxiety and general anxiety scores). There was a positive correlation between negative flying probability estimates and total FAS score, *r*(113) *=* .65, *p* < .001. A positive correlation was also found between negative flying probability estimates and anticipatory flying anxiety, *r*(113) *=* .62, *p* < .001; in-flight anxiety, *r*(113) *=* .69, *p* < .001; and generalised flying anxiety, *r*(113) *=* .46, *p <* .001. The hypothesis was supported.

### Hypotheses 5: Past-Negative, Future and Present-Hedonistic Scores would Positively Predict Probability Estimates for the Occurrence of Negative Flying Events

A standard multiple regression analysis [40] was conducted to explore how the Past-Negative, Present-Hedonistic and Future subscales of the ZTPI uniquely contributed to participants’ estimates of the probability of Flying Negative events as measured by the PS. Table 3 displays the correlations between the predictors and scores on the Probability Scale, the significance value, the standardized regression coefficient (β) and the semi-partial correlation (*sr*^2^). The analysis revealed that the three time orientation measures of Past-Negative, Present-Hedonistic and Future orientation explained 7% of the variance in participants’ estimates of negative flying events occurring *R*^*2*^ = .069, *F*(3, 111) = 2.759, *p* < .05.

**Table 3:** Significance Value, Standardized Regression Coefficient (β) and Semi-Partial Correlations (sr_1_^2^) for Predictors and Estimates of Negative Flying Outcomes (Probability Scale).

	Past-Negative (ZTPI)	Present-Hedonistic (ZTPI)	Future (ZTPI)
**Pearson correlation**	0.182	-0.157	0.007
**Sig. value**	0.025*	0.041*	0.693
**β**	0.211	-0.199	0.034
***sr***^***2***^	0.043	0.036	0.001

* *p* < 0.05

Examination of the beta weights indicated that Past-Negative orientation statistically significantly (positively) predicted Flying Negative scores. Present-Hedonistic orientation statistically significantly (negatively) predicted Flying Negative scores, which was contrary to the hypothesised direction of the relationship. Future orientation did not significantly predict Flying Negative scores. Hypothesis 5 was, therefore, partially supported.

In order to determine whether the relationship between probability estimates and time perspective variables were specific to estimates of negative flying-related events two additional multiple regression analyses were conducted. Past-Negative, Present-Hedonistic and Future ZTPI scores did not significantly predict estimates of General Negative events occurring *F*(4, 110) = 1.858, *p* = .123, *R*^2^ = .063. Similarly, Past-Negative, Present-Hedonistic and Future scores did not significantly predict estimates of General Positive events occurring *F*(4, 110) = .775, *p* = .544, *R*^2^ = .027.

## Discussion

Probability bias pertaining to the likelihood of threat-relevant negative outcomes has been found across a range of anxiety disorders [18, 19, 21]. The present study aimed to investigate if probability bias could be demonstrated in a sample of individuals with high levels of anxiety associated with flying. The results of the present study suggest that the flying phobia group estimated the probability of negative flying-related events occurring as significantly higher than non-flying phobics. This finding provides support for Hypothesis 1 and is, importantly, the first study to demonstrate probability bias in flying phobics.

As noted above, judgements of the likelihood of threat-relevant outcomes are believed to be biased by the use of heuristic rules [17]. The potential operation of the *availability heuristic* [23] would suggest the flying phobic group estimated the probability of flying negative events significantly higher than the non-flying phobic group due to the ease with which they could recall threat-relevant memories such as negative flying experiences (where they believed they were in immediate danger) or recalled information consistent with the flying experience being dangerous (e.g. news reports of plane crashes). Similarly, the *simulation heuristic* may be hypothesised to have contributed towards this group difference in estimates, and would suggest that expectancies are influenced by the relative ease of imagining mental simulations of related events [13]. If the simulation heuristic did, at least in part, account for this group difference, then it might be argued that the flying phobic group demonstrated a general predisposition towards more easily generating episodic future thought (autonoetic consciousness, discussed further below) or individuals with high levels of flying anxiety have rehearsed future imagery of negative flying outcomes (e.g. in-flight crashes or engine failure described in the PS) many more times than those with low flying anxiety and, therefore, more readily generate such imagery. Future research should endeavour to explore the extent to which episodic future thought or memories influence probability estimates in flying phobia and whether the availability heuristic at least partially explains this probability bias.

The hypothesis that probability estimates regarding negative flying-related outcomes would be positively associated with participant anxiety experienced with flying (including anxiety experienced in anticipation of flying, in-flight anxiety and general flying-related anxiety) was supported. The existence of a probability bias in flying phobia, and its relationship to the level of anxiety experienced when flying, is consistent with cognitive behavioural theory suggesting that anxiety associated with a given stimulus or scenario is proportionate to the perceived likelihood of negative outcomes and that anxiety disorders reflect the overestimation of threat [41]. The demonstration of this probability bias suggests a clear need for clinicians and researchers to measure and address this bias within the treatment of flying phobia. Indeed, it would be useful to assess whether the psychoeducation component of psychological treatments of flying phobia [42] satisfactorily addresses this bias.

Based upon previous research [21] it was hypothesised that there would be no difference between ratings made by flying and non-flying phobics on General Positive events. This hypothesis was supported. However, contrary to Hypothesis 2, there was a significant difference between the ratings made by flying phobics and non-flying phobics regarding the probability of General Negative events occurring. Flying phobics estimated the probability of General Negative events as being significantly higher than non-flying phobics. This unpredicted difference may be due to comorbid anxiety or depression in the flying phobic condition, which the present study did not assess and, thus, may be considered a limitation. Öst and Csatlsos [21] administered the Beck Anxiety Inventory (BAI [43]) and the Beck Depression Inventory (BDI [44]) to participants and found that BAI scores correlated significantly with the probability ratings on the PS of both claustrophobic and negative events, whilst BDI scores correlated with the probability ratings of negative events. Moreover, research has found that participants with depression typically make higher estimates of the likelihood of negative events occurring to themselves in the future [45]. Anxiety disorders and depression are highly comorbid and specific phobias in particular are strongly associated with other anxiety and mood disorders [46–47]. These studies suggest that the flying phobics in the present study may have also been experiencing depressive symptoms at the time of participation, which contributed to higher estimates of the probability of general negative events. An alternative interpretation of these findings is that individuals who develop flying phobia are more susceptible to imagining negative or catastrophic future outcomes, which speaks to the construct of autonoetic consciousness.

Finally, the present study aimed to investigate whether time orientation (measured by three subscales of the ZTPI) predicted the perceived likelihood of negative flying-related outcomes. It was hypothesised that all three subscales would positively predict estimates of the probability of negative flying events. Surprisingly, Future and Present-Hedonistic subscales did not positively predict probability estimates. In addition, contrary to expectations, Present-Hedonistic orientation made a statistically significant negative contribution to the prediction of Flying Negative scores (i.e. higher levels of Present-Hedonistic scores were associated with lower estimates of the probability of negative flying outcomes). Scores on the Past-Negative subscale did make a statistically significant contribution to the prediction of Flying Negative scores in the hypothesised direction; however, the combined model, though statistically significant, explained only 7% of the variance in probability estimates.

Higher scores on the Future and Present-Hedonistic subscales of the ZTPI have been found to be significant predictors of mental time travel (autonoetic consciousness) and a temporal orientation towards the future was hypothesised to influence estimates of the likelihood of the occurrence of negative flying-related future events. This prediction was not supported. A simple interpretation of this finding may be that Future and Present-Hedonistic temporal orientations do not predict the manner (e.g. amount or vividness of mental imagery) in which individuals envisage future events or individual predictions of the probability of such events occurring. Additionally, the PS asked the participants to envisage scenarios that would be conceptualised as threatening outcomes for flying phobics and it must be considered that flying phobics may have avoided engaging in envisaging such scenarios when making probability estimates. Indeed, there is a precedence within the anxiety disorder literature to suggest that threat-related future imagery may be avoided or suppressed. For example, individuals with GAD have been found to generate less detailed episodic future thoughts relative to normal controls [48], and it has been suggested that they engage in cognitive avoidance and suppression of threatening future-oriented mental imagery [49]. Clearly, utilising a direct measure of the presence or absence of mental imagery whilst participants engage in making probability estimates (in addition to measuring temporal orientation) would have significantly improve the present study’s methodology and must therefore be considered a priority for future research

The findings relating to Past-Negative orientation may suggest that a negative temporal orientation towards the past, which will incorporate a tendency to recall aversive past events, may impact on the perceived likelihood of future events. Clark and Rock [35] proposed that the retrieval and reliving of aversive flight experiences and anxious states during previous plane flights may serve to perpetuate anxiety and perceived threat in flying phobics and retrieval of episodic memories has been demonstrated to influence the manner in which people imagine future events [50]. As noted above, the selective retrieval of threat-relevant outcomes may increase the perceived likelihood of threat-relevant outcomes occurring [36], and perhaps participants’ ratings of the likelihood of negative flying outcomes were influenced by their recall of negative flying experiences (whether personal or vicarious).

An alternative interpretation of this finding is that because the Past-Negative subscale measures a generally negative view of one’s past and is negatively associated with psychological well-being and predicted life satisfaction [31], this temporal orientation may simply predispose people to think bad things will happen in the future. Additionally, the fact that participants knew they were participating in a flying phobia study may have primed them to recall or anticipate negative outcomes pertaining to flying. In contrast, because the Present-Hedonistic subscale (an orientation towards present-focussed enjoyment) has been found to be positively associated with psychological well-being, current and predicted-future life satisfaction [31], this temporal orientation may predispose people to think bad things are unlikely to happen in the future.

The results of the present study suggest that further research into the role of temporal orientation and probability bias in flying phobia is warranted. Given that both temporal orientation and the perceived probability of future events may involve mental imagery and autonoetic consciousness, future extensions of this research should employ targeted measures of both of these variables. Such targeted measurement would help to inform an understanding of whether episodic future thought or recall of negative flying related experiences influences individual judgments of the likelihood of negative flying-related events occurring.

In considering the study’s limitations it should be cautioned that although referred to as a “flying phobic” group, individuals in this group did not undergo any diagnostic evaluation beyond reporting high levels of anxiety when flying. Therefore, it is unclear to what degree the sample would be comparable to those with a DSM-5 [1] diagnosis of specific phobia. A further limitation is that the PS described external negative flying-related outcomes and many individuals with flying phobia report that their primary flying-related fear centres upon internal threat (e.g. the consequences of panic symptoms and loss of control when flying [7]). Consequently, it is not clear that the flying-related negative outcomes assessed reflected participants’ primary fear. Finally, this was an internet-based study and, thus, sampling concerns exist. Relatively little is known about the characteristics of individuals in online communities and there have been mixed findings about the generalisability and representativeness of online populations [51].

Despite the aforementioned limitations, the present study found a significant and robust difference in the probability estimates between the flying phobic and non-flying phobic groups for negative outcomes related to flying. The present study, therefore, extends previous research in being the first to demonstrate a probability bias regarding negative flying-related outcomes in a sample of flying phobics. Whilst it is well documented that anxiety is a state of anticipatory apprehension over possible negative occurrences in the future, this is the first study that attempted to measure the relationship between the perceived probability of fear-relevant outcomes occurring and time orientation. The findings indicated that a Past-Negative orientation, indicating a negative relationship with one’s past and memories, is associated with increased estimates of the probability of negative flying outcomes. In contrast, a Present-Hedonistic orientation was negatively associated with such estimates. Further research is needed to establish the factors which contribute to probability estimates regarding fear-relevant outcomes in flying phobia and whether time orientation, mental imagery and autonoetic consciousness plays a part in such estimates.

## Supporting Information

S1 File Data file. (SAV)
